# The Dynamics and Optimal Control of a Hand-Foot-Mouth Disease Model

**DOI:** 10.1155/2018/9254794

**Published:** 2018-07-05

**Authors:** Hongwu Tan, Hui Cao

**Affiliations:** Department of Mathematics, Shaanxi University of Science & Technology, Xi'an 710021, China

## Abstract

We build and study the transmission dynamics of a hand-foot-mouth disease model with vaccination. The reproduction number is given, the existence of equilibria is obtained, and the global stability of disease-free equilibrium is proved by constructing the Lyapunov function. We also apply optimal control theory to the hand-foot-mouth disease model. The treatment and vaccination interventions are considered in the hand-foot-mouth disease model, and the optimal control strategies based on minimizing the cost of intervention and minimizing the number of the infected people are given. Numerical results show the usefulness of the optimization strategies.

## 1. Introduction

Hand, foot, and mouth disease (HFMD) is a common infectious disease caused by a group of viruses known as enteroviruses (EVs) [1, 2]. HFMD usually affects children, typically affecting children who are less than 10 years, but it can also affect adults [2]. However, adults are immune to the disease due to the antibodies in their bodies, although most of them are exposed to the virus [3].

The virus of HFMD spreads easily through coughing, sneezing, and infected stool. It usually takes 3 − 7 days for a person to get symptoms of HFMD disease after being exposed to the virus of HFMD. This is called the incubation period of HFMD. Although many HFMD infected people remain asymptomatic, the symptoms of HFMD include sores in or on the mouth and on the hands, feet, and sometimes the buttocks and legs. The sores may be painful, and these sores may be eased with the use of medication [4]. In fact, there is no specific treatment for HFMD, and many doctors do not issue medicine for this illness since HFMD is a viral disease that has to run its course [5].

Numerous large outbreaks of HFMD have occurred in many areas of the world, such as the United States of America, Australia, Malaysia, Japan, and China since 1997, which have caused death and neurological sequelae, and have become a growing public health threat [6–10]. Fortunately, Chinese scientists have developed the first vaccine to protect children against enterovirus 71, or EV71, that causes the common and sometimes deadly HFMD in 2013 [11]. However, the literature on the mathematical modeling of the transmission of HFMD is rather scant. In particular, there are fewer literatures on mathematical models of HFMD with vaccination. Urashima et al. and Wang and Sung tried to find the relationship between the outbreak of HFMD with the weather patterns in Taiwan and Tokyo, respectively [12, 13]. Tiing and Labadin used a deterministic SIR model to predict the number of infected cases and the duration of an outbreak when it occurs in Sarawak [14]. Roy and Halder proposed a deterministic SEIR model of HFMD and did numerical simulations [15]. Liu and Yang et al. used the SEIQRS model to take into account the quarantine measure [5, 16]. Recently, Samanta discussed a delay HFMD model with pulse vaccination strategy [2].

In this paper, we only consider the children below the age of 10 years since the children above the age of 10 years are immune to the disease because their immune systems are relatively perfect. The aim of our study is to use mathematical modeling to gain some insights into the transmission dynamics of HFMD when the population is vaccinated. The paper is organized as follows. In Section 2, we formulate the HFMD model with vaccination and define the basic reproduction number. In Section 3, we obtain the existence of equilibria of model, prove the global stability of disease-free equilibrium, and analyze the global stability of endemic equilibrium of model by constructing the Lyapunov function. In Section 4, we discuss the optimal control problem by adding two control functions. At last, we display the numerical simulation and give the conclusion.

## 2. Model Formulation

Enteroviruses (EVs) that are most frequently reported as causing HFMD outbreaks include enterovirus 71 (EV71) and coxsackievirus A16 (CVA16). Other human enteroviruses serotypes, such as CVA4, CVA5, CVA6, and CVA10, have also been reported in cases of HFMD [1]. Because only EV71 vaccine was on market which could prevent the HFMD induced by EV71 infection, we will consider dividing the infectious individuals into two classes, which are infectious individuals *I*_1_ infected with EV71 and infectious individuals *I*_2_ infected with CVA16 or other human enteroviruses serotypes.

Let *N*(*t*) be total number of children below the age of 10 years at time *t*. We divide children below the age of 10 years into five compartments, including susceptible individuals *S*(*t*), latent individuals *E*(*t*), infectious individuals *I*_1_(*t*) and *I*_2_(*t*), vaccination individuals *V*(*t*), and recovery individuals *T*(*t*). It is clear that *N*(*t*) = *S*(*t*) + *E*(*t*) + *I*_1_(*t*) + *I*_2_(*t*) + *V*(*t*) + *T*(*t*). The dynamical model for HFMD transmission in children below the age of 10 years is in the following:

(1) dStdt=1−pb−β1StI1t−β2StI2t−μ+ωSt+η1Vt+η2Tt,dEtdt=β1StI1t+β2StI2t−μ+αEt,dI1tdt=qαEt−μ+d1+γ1I1t,dI2tdt=1−qαEt−μ+d2+γ2I2t,dVtdt=pb−μ+ω+η1Vt,dTtdt=γ1I1t+γ2I2t−μ+ω+η2Tt,

where *b* > 0 is the birth rate of the population; *p* ≥ 0 is the vaccine rate of the population; *β*_1_ > 0 is the transmission coefficient of the infectious individuals infected with EV71; *β*_2_ > 0 is the transmission coefficient of the infectious individuals infected with CVA16; *μ* > 0 is the natural death rate; *α* > 0 is the per-capita rate of the progression from latent individuals to infectious individuals; *q* ≥ 0 is the percentage of individuals infected with EV71 from latent individuals to infectious individuals; accordingly, 0 < 1 − *q* < 1 is the percentage of individuals infected with CVA16 or other human enteroviruses serotypes from latent individuals to infectious individuals; *d*_1_, *d*_2_ > 0 is the disease induced death rate of infectious individuals *I*_1_, *I*_2_, respectively; *γ*_1_, *γ*_2_ ≥ 0 is the treatment rate of the infectious individuals *I*_1_, *I*_2_, respectively; *ω* ≥ 0 is the removal rate of population; *η*_1_ ≥ 0 and *η*_2_ ≥ 0 are the loss of immunity rate of vaccination individuals and recovery individuals, respectively.

In our paper, in order to make the qualitative mathematical analysis, let *β* = *β*_1_ = *β*_2_, *γ* = *γ*_1_ = *γ*_2_, *d* = *d*_1_ = *d*_2_, and *I*(*t*) = *I*_1_(*t*) + *I*_2_(*t*); we simplify model (1) to the following model:

(2) dStdt=1−pb−βStIt−μ+ωSt+η1Vt+η2Tt,dEtdt=βStIt−μ+αEt,dItdt=αEt−μ+d+γIt,dVtdt=pb−μ+ω+η1Vt,dTtdt=γIt−μ+ω+η2Tt.

In the next section, we will discuss dynamics of system (2). It is obvious that any solution of system (2) with nonnegative initial values is nonnegative.

**Lemma 1 .**

Every forward solution (*S*(*t*), *E*(*t*), *I*(*t*), *V*(*t*), *T*(*t*)) of system (2) eventually enters *Ω* = {(*S*, *E*, *I*, *V*, *T*) ∈ *R*_5_^+^∣*S* + *E* + *I* + *V* + *T* ≤ *b*/*μ*}, and *Ω* is a positively invariant set for (2).

**Proof**

By using *N*(*t*) = *S*(*t*) + *E*(*t*) + *I*(*t*) + *V*(*t*) + *T*(*t*), from system (2), we have

(3) dNtdt=b−μNt−dIt−ωSt+Vt+Tt≤b−μNt.

It is obvious that lim sup_*t*→+*∞*_*N*(*t*) ≤ *b*/*μ*, which implies that *N*(*t*) ≤ *b*/*μ*. That is, every solution of system (2) eventually enters *Ω*, and *Ω* is positively invariant with respect to system (2). This proves the lemma.

The dynamics of system (2) will be investigated in the following bounded feasible region:

(4) $$\begin{array}{c} \Omega =\left\{\;\left(\;S,E,I,V,T\;\right)\in R_{5}^{+}\;|\;S+E+I+V+T\leq \frac{b}{\mu }\;\right\}. \end{array}$$

Using the relation *V*(*t*) = *N*(*t*) − *S*(*t*) − *E*(*t*) − *I*(*t*) − *T*(*t*), we may reduce system (2) to the following equivalent system:

(5) dStdt=Λ−βStIt−μ+ωSt+η2Tt,dEtdt=βStIt−μ+αEt,dItdt=αEt−μ+d+γIt,dTtdt=γIt−μ+ω+η2Tt,

with Λ = (1 − *p*)*b* + *pbη*_1_/(*μ* + *ω* + *η*_1_), on the positively invariant set

(6) $$\begin{array}{c} \bar{\Omega }=\left\{\;\left(\;S,E,I,T\;\right)\in R_{4}^{+}\;|\;S+E+I+T<\frac{b}{\mu }\;\right\}. \end{array}$$

In the following, since system (5) has the same dynamic as (2), we will discuss the dynamic of system (5) on $\bar{\Omega }$.

Following van den Diessche and Watmough [17, 18], we can obtain the basic reproduction number:

(7) R0=αβμ+αμ+d+γ×1−pbμ+ω+η1+pbη1μ+ωμ+ω+η1=αβμ+αμ+d+γ×Λμ+ω.

Each term in *R*_0_ has clear epidemiological interpretation. *α*/(*μ* + *α*) is the proportion that latent individuals progress to infectious class. 1/(*μ* + *d* + *r*) is the average infectious period. Λ/(*μ* + *ω*) is the total amount of population in the case that the infected individuals in population do not exist. Thus, *αβ*/(*μ* + *α*)(*μ* + *d* + *γ*) × Λ/(*μ* + *ω*) are average new cases generated by a typical infectious member in the entire infection period.

The basic reproduction number *R*_0_, for model (2) in the absence of controls, i.e., in the case *p* = *γ* = 0, which means that model (2) does not have vaccination individuals *V*(*t*) and recovery individuals *R*(*t*), is proportional to the transmission coefficient *β* and is given by

(8) $$\begin{array}{c} \begin{array}{c} {\left.R_{0}\right|}_{p=\gamma =\eta_{1}=0}=\frac{\alpha \beta }{\left(\mu +\alpha \right)\left(\mu +d\right)}\times \frac{b}{\left(\mu +\omega \right)}. \end{array} \end{array}$$

It is clear that

(9) R0−R0p=γ=η1=0=bαβμ+αμ+ω×−pμ+dμ+ω−γμ+ω+η1μ+d+γμ+ω+η1μ+d≤0,

which implies that the vaccination and treatment have contributed to decrease of the *R*_0_. That is, the vaccination and treatment help to slow down the HFMD spread.

Three parameters have a high impact on *R*_0_: *p* and *γ* decrease *R*_0_, respectively, and *β* increases *R*_0_.

## 3. The Existence and Stability of Equilibria

We first discuss the existence of equilibria of system (5). Directly calculating system (5), we obtain the disease-free equilibrium *P*^0^ = (*S*^0^, *E*^0^, *I*^0^, *T*^0^), where *S*^0^ = Λ/(*μ* + *ω*), and *E*^0^ = *I*^0^ = *T*^0^ = 0. In addition, there exists a endemic equilibrium *P*^*∗*^ = (*S*^*∗*^, *E*^*∗*^, *I*^*∗*^, *T*^*∗*^) when *R*_0_ > 1, where

(10) S∗=μ+αμ+d+γαβ,E∗=μ+d+γI∗α,T∗=γI∗μ+ω+η2,I∗=μ+ωμ+ω+η2μ+αμ+d+γμ+ω+η2−αη2γ×μ+αμ+d+γβR0−1.

Summarizing the above discussion, we can obtain the following result.

**Theorem 2 .**

If *R*_0_ ≤ 1, system (5) has only the disease-free equilibrium *P*^0^ = (*S*^0^, *E*^0^, *I*^0^, *T*^0^) = (Λ/(*μ* + *ω*), 0,0, 0). If *R*_0_ > 1, besides the disease-free equilibrium *P*^0^, system (5) also has a endemic equilibrium *P*^*∗*^ = (*S*^*∗*^, *E*^*∗*^, *I*^*∗*^, *T*^*∗*^).

In the following, we will discuss the stability of equilibria of system (5). The stability of disease-free equilibrium of system (5) firstly was proved.

**Theorem 3 .**

If *R*_0_ ≤ 1, the disease-free equilibrium *P*^0^ of system (5) is globally asymptotically stable, while if *R*_0_ > 1, the disease-free equilibrium *P*^0^ of system (5) is unstable.

**Proof**

The Jacobian matrix of system (5) at the disease-free equilibrium *P*^0^ is

(11) J0=−μ+ω0−βS0η20−μ+αβS000α−μ+d+γ000γ−μ+ω+η2.

It is clear that *λ*_1_ = −(*μ* + *ω*) < 0 and *λ*_2_ = −(*μ* + *ω* + *η*_2_) < 0 are the eigenvalues of matrix *J*^0^. The rest of the eigenvalues of matrix *J*^0^ satisfy the following equation:

(12) λ2+μ+α+μ+d+γλ+μ+αμ+d+γ1−R0=0.

Obviously,

(13) Δ=μ+α+μ+d+γ2−4μ+αμ+d+γ1−R0=μ+α−μ+d+γ2+4μ+αμ+d+γR0>0.

It implies that (12) has two real roots, *λ*_3_ and *λ*_4_, which satisfy

(14) λ3+λ4=−μ+α+μ+d+γ<0,λ3λ4=μ+αμ+d+γ1−R0.

If *R*_0_ < 1, we have *λ*_3_*λ*_4_ > 0, which implies that the real parts of *λ*_3_ and *λ*_4_ are both negative. That is, the disease-free equilibrium *P*^0^ is locally asymptotically stable. Meanwhile if *R*_0_ > 1, we obtain *λ*_3_*λ*_4_ < 0. It implies that the real part of *λ*_3_ or the real part of *λ*_4_ is positive. Therefore, *P*^0^ is unstable.

For the critical case *R*_0_ = 1, the Jacobian matrix *J*^0^ has three negative real eigenvalues −(*μ* + *ω*), −(*μ* + *ω* + *η*_2_), and −(*μ* + *α* + *μ* + *d* + *γ*), and one zero eigenvalue.

We introduce the matrix of eigenvectors

(15) $$\begin{array}{c} P=\left(\begin{array}{cccc} \frac{\mu +\alpha }{\mu +\omega }\left(\frac{\gamma \eta_{2}}{\mu +\omega +\eta_{2}}-\beta S^{0}\right) & 1 & -1 & \frac{-\beta S^{0}p_{1}+\eta_{2}\gamma }{\omega -\left(\mu +\alpha +d+\gamma \right)}\\ \beta S^{0} & 0 & 0 & \frac{-\left(\mu +\alpha \right)p_{1}}{\alpha }\\ \mu +\alpha & 0 & 0 & p_{34}\\ \frac{\left(\mu +\alpha \right)\gamma }{\mu +\omega +\eta_{2}} & 0 & 1 & \gamma \end{array}\right) \end{array}$$

with *p*_34_ = (*μ* + *α* + *d* + *γ*)−(*ω* + *η*_2_), such that *J*^0^*P* = *PA*, where

(16) A=00000−μ+ω0000−μ+ω+η20000−μ+α+μ+d+γ.

We make the linear transformation (*u*, *v*, *w*, *x*)^*T*^ = *P*^−1^(*S* − *S*^0^, *E*, *I*, *T*)^*T*^, where

(17) $$\begin{array}{c} P^{-1}=\left(\begin{array}{cccc} 0 & \frac{\alpha }{\left(\mu +\alpha +\mu +d+\gamma \right)\left(\mu +\alpha \right)} & \frac{1}{\mu +\alpha +\mu +d+\gamma } & 0\\ 1 & p_{22}^{-1} & p_{23}^{-1} & 1\\ 0 & \frac{\gamma \alpha \left(\mu +\omega +\eta_{2}-p_{1}\right)}{p_{1}\left(\mu +d+\gamma +\mu +\alpha \right)} & -\frac{{\left(\mu +\alpha \right)}^{2}p_{1}\gamma }{\alpha \left(\mu +\omega +\eta_{2}\right)}-\beta S^{0}\gamma & 1\\ 0 & \frac{-\alpha }{\left(\mu +\alpha +\mu +d+\gamma \right)p_{1}} & \frac{\mu +d+\gamma }{\left(\mu +\alpha +\mu +d+\gamma \right)p_{1}} & 0 \end{array}\right) \end{array}$$

with *p*_22_^−1^ = *γα*(*μ* + *ω* + *η*_2_ − *p*_1_)/*p*_1_(*μ* + *d* + *γ* + *μ* + *α*) + (*μ* + *α*)(*η*_2_*γ* − *βS*^0^*p*_1_)/(*ω* − *μ* − *α* − *d* − *γ*) + ((*μ* + *α*)^2^*p*_1_/ *α*(*μ* + *ω*))(*γη*_2_/(*μ* + *ω* + *η*_2_) − *βS*^0^), *p*_23_^−1^ = −*βS*^0^*γ* − ((*μ* + *α*)^2^*p*_1_/(*μ* + *ω*)*α*)(*γ* − *βS*^0^) − *βS*^0^(*η*_2_*γ* − *βS*^0^*p*_1_)/(*ω* − *μ* − *α* − *d* − *γ*).

The Jacobian matrix for the differential equations of (*u*, *v*, *w*, *x*) about the zero equilibrium is exactly *A*. To analyze the local asymptotic stability of this zero equilibrium, we need to calculate the restricted dynamical system on the center manifold for *u* sufficiently small and *v* = *O*(*u*^2^), *w* = *O*(*u*^2^), *x* = *O*(*u*^2^) [19]. Note that *u* = *αE*/(*μ* + *α* + *μ* + *d* + *γ*)(*μ* + *α*) + *I*/(*μ* + *α* + *μ* + *d* + *γ*); from the second, third, and forth equations of system (5), we obtain

(18) $$\begin{array}{c} \left(\;\mu +\alpha +\mu +d+\gamma \;\right)\left(\;\mu +\alpha \;\right)u^{\prime }=\alpha \beta \left(\;S-S^{0}\;\right)I. \end{array}$$

Next, we make use of *S* − *S*^0^ = (((*μ* + *α*)/(*μ* + *ω*))(*γη*_2_/(*μ* + *ω* + *η*_2_) − *βS*^0^))*u* + *O*(*u*^2^), *E* = *βS*^0^*u* + *O*(*u*^2^), *I* = (*μ* + *α*)*u* + *O*(*u*^2^), and *T* = (*μ* + *α*)*γu*/(*μ* + *ω* + *η*_2_) + *O*(*u*^2^) to obtain

(19) μ+α+μ+d+γu′=−αβμ+αμ+ω×μ+αμ+d+γμ+ω+η2−αγη2αμ+ω+η2u2+Ou3.

Since restricted system (19) is stable about *u* = 0, original system (5) is locally stable about the disease-free equilibrium *P*^0^ when *R*_0_ = 1.

In the following, we study the global stability when *R*_0_ ≤ 1. Let *L*(*E*(*t*), *I*(*t*)) = *E*(*t*)+((*μ* + *α*)/*α*)*I*(*t*); we have

(20) dLtdt=dEtdt+μ+ααdItdt=βStIt−μ+ααμ+d+γIt≤βS0−μ+ααμ+d+γIt=μ+αμ+d+γαR0−1It≤βS0−μ+ααμ+d+γIt=μ+αμ+d+γαR0−1It≤0.

Furthermore, *dL*/*dt* = 0 if and only if *I* = 0 or *R*_0_ = 1. Therefore, the largest compact invariant set in $\left\{(S,E,I,T)\in \bar{\Omega }:dL/dt=0\right\}$ is the singleton {*P*^0^}. LaSalle's invariance principle [20] then implies that *P*^0^ is globally stable in $\bar{\Omega }$.

Next, we discuss the global asymptotical stability of the endemic equilibrium of system (5). The local stability of the endemic equilibrium firstly was discussed, and the global stability of the endemic equilibrium also was discussed by constructing the Lyapunov function.

**Theorem 4 .**

If *R*_0_ > 1, the endemic equilibrium *P*^*∗*^ of system (5) is locally asymptotically stable.

**Proof**

The Jacobian matrix of system (5) at the endemic equilibrium *P*^*∗*^ is

(21) $$\begin{array}{c} J^{*}=\left(\begin{array}{cccc} -\beta I^{*}-\left(\mu +\omega \right) & 0 & -\beta S^{*} & \eta_{2}\\ \beta I^{*} & -\left(\mu +\alpha \right) & \beta S^{*} & 0\\ 0 & \alpha & -\left(\mu +d+\gamma \right) & 0\\ 0 & 0 & \gamma & -\left(\mu +\omega +\eta_{2}\right) \end{array}\right). \end{array}$$

It is clear that the eigenvalues of matrix *J*^*∗*^ satisfy the following equation:

(22) $$\begin{array}{c} \lambda^{4}+A_{1}\lambda^{3}+A_{2}\lambda^{2}+A_{3}\lambda +A_{4}=0, \end{array}$$

where

(23) A1=μ+ω+η2+βI∗+μ+ω+μ+α+μ+d+γ,A2=μ+ω+η2βI∗+μ+ω+μ+ω+η2+βI∗+μ+ωμ+α+μ+d+γ,A3=μ+ω+η2βI∗+μ+ωμ+α+μ+d+γ+μ+αμ+d+γβI∗,A4=βI∗μ+ω+η2μ+αμ+d+γ−αγη2.

By directly calculating, we have

(24) Ai>0,i=1,2,3,4,D1=A1>0,D2=A1A31A2=A1A2−A3=μ+ω+η2+βI∗+μ+ωA2+μ+α+μ+d+γμ+ω+η2·μ+α+μ+d+γ+βI∗+μ+ωμ+α+μ+d+γ2βI∗+μ+ω+μ+αμ+ωμ+d+γ>0,D3=A1A301A2A40A1A3=A3D2−A12A4=A3μ+ω+η2βI∗+μ+ω·μ+ω+η2+βI∗+μ+ω+βI∗+μ+ω·μ+d+γ2+μ+αμ+ωμ+d+γ+βI∗μ+ω+η2μ+α2μ+ω+η2+βI∗+μ+ωA1+βI∗αγη2A12+μ+ω+η2μ+α+μ+d+γμ+ω+η2+βI∗+μ+ω2·βI∗+μ+ωμ+d+γ+μ+ωμ+α+μ+ωμ+ω+η2μ+α+μ+d+γ2μ+ω+η2μ+α+μ+d+γ+μ+αβI∗+μ+ω+βI∗μ+ω+η2μ+d+γμ+α+μ+d+γ2+βI∗μ+αμ+d+γβI∗+μ+ωμ+α+μ+d+γ·μ+ω+η2+βI∗+μ+ω+μ+α>0,D4=A1A3001A2A400A1A3001A2A4=A1A2A3A4−A12A42−A32A4=A4D3>0,

The Routh-Hurwitz criterion [21] implies that all eigenvalues of characteristic equation (22) have negative real part; that is, *P*^*∗*^ is locally asymptotically stable when *R*_0_ > 1.

**Figure 1:**
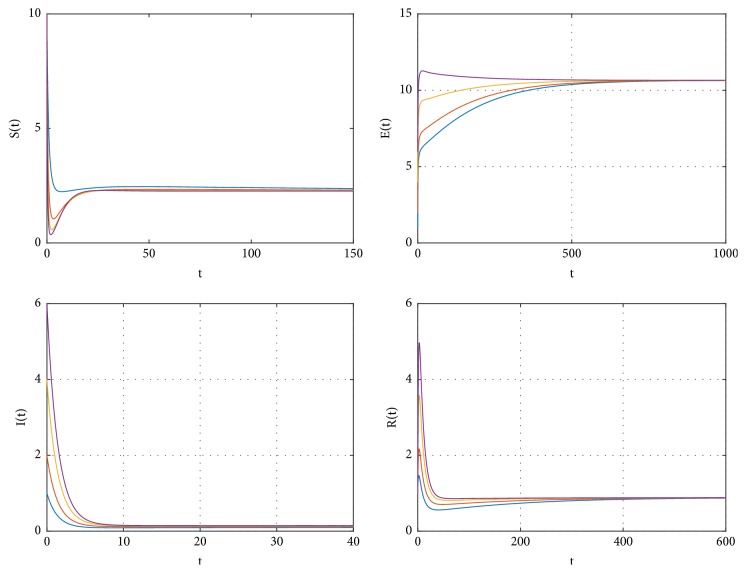
The global stability of *P*^*∗*^ of system (2) when *R*_0_ > 1.

It is difficult to show the global stability of endemic equilibrium *P*^*∗*^ by the theoretical methods. We will use the numerical simulation to display the global stability of endemic equilibrium *P*^*∗*^; see Figure 1. The parameters are taken to be *p* = 0.8, *b* = 1, *β* = 0.5, *μ* = 0.006, *ω* = 1/12, *η*_1_ = 0.02, *η*_2_ = 0.03, *α* = 0.01, *d* = 0.002, and *γ* = 0.7, respectively. Accordingly, the basic reproduction number *R*_0_ = 1.7112 > 1. The simulation demonstrates that endemic equilibrium *P*^*∗*^ may be globally stable when *R*_0_ > 1.

## 4. The HFMD Model with Optimal Controls

In this section, we present the optimal control problem by adding to the model (2) two control functions *u*_1_(*t*) and *u*_2_(*t*). The HFMD model with controls is given by the following equations:

(25) dStdt=1−u1tb−βStIt−μ+ωSt+η1Vt+η2Tt,dEtdt=βStIt−μ+αEt,dItdt=αEt−μ+d+u2tIt,dVtdt=u1tb−μ+ω+η1Vt,dTtdt=u2tIt−μ+ω+η2Tt.

The aim is to find the optimal values *u*_1_^*∗*^ and *u*_2_^*∗*^ of the controls *u*_1_ and *u*_2_, such that the associated state trajectories *S*(*t*), *E*(*t*), *I*(*t*), *V*(*t*), and *T*(*t*) are solution of system (25) in the time interval [0, *t*_*f*_] with initial conditions *S*(0), *E*(0), *I*(0), *V*(0), and *T*(0) and minimize the objective functional. Here the objective functional considers the number of latent individuals *E*, the number of the infectious individuals *I*, and the implementation cost of the strategies associated with the controls *u*_*i*_, *i* = 1,2. The controls are bounded between 0 and 1.

We consider state system (25) of ordinary differential equations in *R*^5^ with the set of admissible control functions given by

(26) U=u1t,u2t∈L∞0,tf2 ∣ 0≤u1t,u2t≤1,  ∀t∈0,tf.

The objective functional is given by

(27) Ju1t,u2t=∫0tfEt+It+B12u12t+B22u22tdt,

where the constants *B*_1_ and *B*_2_ are a measure of the relative cost of the interventions associated with the controls *u*_1_ and *u*_2_, respectively.

We consider the optimal control problem of determining (*S*(*t*), *E*(*t*), *I*(*t*), *V*(*t*), *T*(*t*)), associated with an admissible control pair (*u*_1_^*∗*^, *u*_2_^*∗*^) ∈ *U* on the time interval [0, *t*_*f*_], satisfying (25) and the initial conditions *S*(0), *E*(0), *I*(0), *V*(0), and *T*(0) and minimizing cost functional (27); that is,

(28) $$\begin{array}{c} J\left(\;u_{1}^{*},u_{2}^{*}\;\right)=\min\left\{\;J\left(\;u_{1},u_{2}\;\right)\;|\;\left(\;u_{1},u_{2}\;\right)\in U\;\right\}. \end{array}$$

**Theorem 5 .**

There exists an optimal control pair (*u*_1_^*∗*^, *u*_1_^*∗*^) such that

(29) $$\begin{array}{c} J\left(\;u_{1}^{*},u_{2}^{*}\;\right)=\min\left\{\;J\left(\;u_{1},u_{2}\;\right)\;|\;\left(\;u_{1},u_{2}\;\right)\in U\;\right\}, \end{array}$$

subject to state system (25) with initial conditions *S*(0), *E*(0), *I*(0), *V*(0), and *T*(0).

**Proof**

The integrand of the objective functional *J* given by (27) is convex on the closed, convex control set *U*. The conditions for the existence of optimal control are satisfied as the model is linear in the control variables and is bounded by a linear system in the state variables [22].

According to the Pontryagin Maximum Principle [23], we now derive the necessary conditions that a pair of optimal controls and corresponding states must satisfy. To this purpose, we define the Hamiltonian function for the system:

(30) H=E+I+B12u12+B22u22+λ1dSdt+λ2dEdt+λ3dIdt+λ4dVdt+λ5dTdt,

where *λ*_*i*_, *i* = 1,2, 3,4, 5, are the adjoint variables.

**Theorem 6 .**

Given an optimal control *u*^*∗*^ = (*u*_1_^*∗*^, *u*_2_^*∗*^) on [0, *t*_*f*_] and corresponding state solution (*S*(*t*), *E*(*t*), *I*(*t*), *V*(*t*), *T*(*t*)) of corresponding state system (25) with initial conditions *S*(0), *E*(0), *I*(0), *V*(0), and *T*(0), there exist adjoint variables *λ*_*i*_, *i* = 1,2, 3,4, 5, satisfying

(31) dλ1tdt=λ1−λ2βI+λ1μ+ω,dλ2tdt=λ2−λ3α+λ2μ−1,dλ3tdt=λ1−λ2βS+λ3−λ5u2+λ3μ+d−1,dλ4tdt=λ4−λ1η1+λ4μ+ω,dλ5tdt=λ5−λ1η2+λ5μ+ω.

with transversality conditions (or boundary conditions) being

(32) $$\begin{array}{c} \lambda_{i}\left(\;t_{f}\;\right)=0,\;for\;\;i=1,2,3,4,5. \end{array}$$

Furthermore, the optimal controls *u*_1_^*∗*^ and *u*_2_^*∗*^ are given by

(33) u1∗=min⁡max⁡0,λ1−λ4bB1,1,u2∗=min⁡max⁡0,λ5−λ3IB2,1.

**Proof**

The adjoint system results from Pontryagin's Principle [23]:

(34) λ1′t=−∂H∂S,λ2′t=−∂H∂E,λ3′t=−∂H∂I,λ4′t=−∂H∂V,λ5′t=−∂H∂T

with zero transversality. To get the characterization of the optimal control given by [23], we solve the equations on the interior of the control set:

(35) $$\begin{array}{c} \frac{\partial H}{\partial u_{i}}=0,\;i=1,2. \end{array}$$

Using bounds on the controls, we obtain the desired characterization.

## 5. Numerical Results and Discussion

In this section, the numerical simulation results of the optimized control measures for HFMD model (25) with vaccination are presented. First, we solve system (25) over the time interval (0, *t*_*f*_] using a forward fourth-order Runge-Kutta scheme and transversality conditions *λ*_*i*_(*t*_*f*_) = 0, *i* = 1,…, 5. Then, system (31) is solved by a backward fourth-order Runge-Kutta scheme using the current iteration solution of (25). The controls are updated by using a convex combination of the previous controls and the values from (33). The iteration is stopped when the values of unknowns at the previous iteration are very close to the ones at the present iteration.

**Figure 2:**
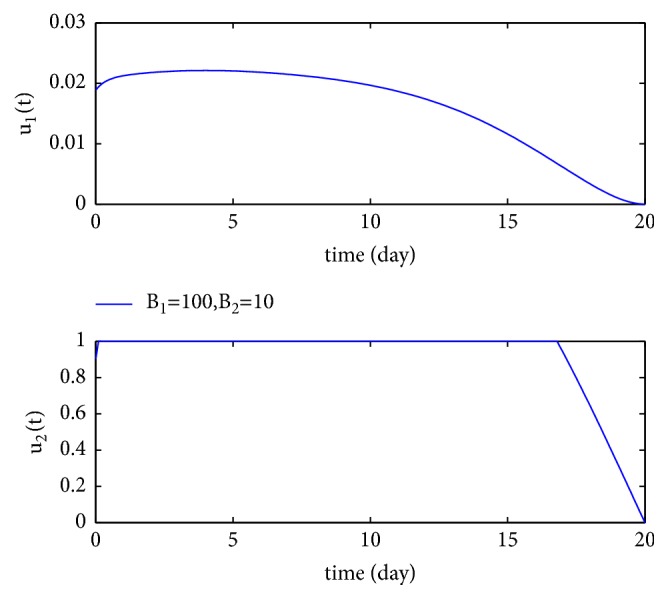
The optimal control variables *u*_1_^*∗*^ and *u*_2_^*∗*^ when *R*_0_ > 1.

**Figure 3:**
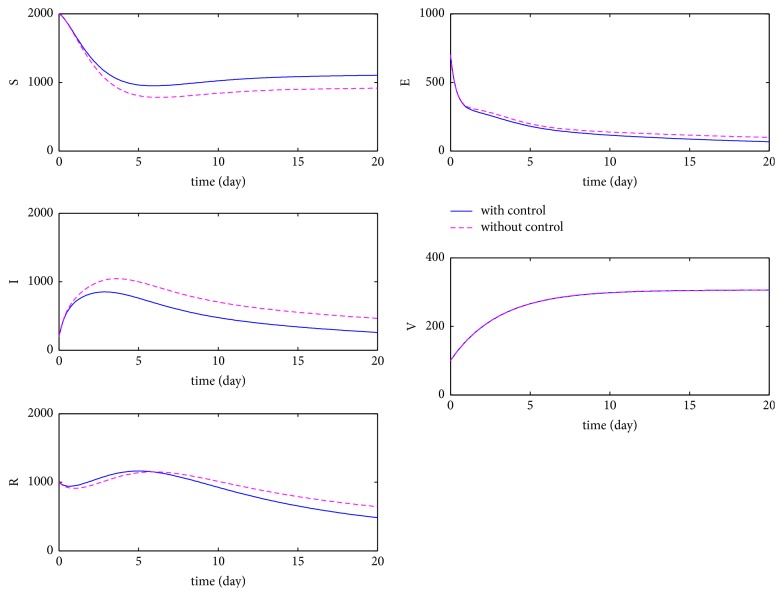
The dynamics of HFMD with and without optimal control when *R*_0_ > 1.

We first compare the number of the susceptible individuals, latent individuals, infectious individuals, vaccination individuals, and the recovery individuals with and without controls, respectively. Take *b* = 2, *p* = 0.5, *β* = 0.04, *μ* = 0.0017, *ω* = 0.125, *η*_1_ = 0.5, *η*_2_ = 0.2, *α* = 1.75, *d* = 0.0034, *γ* = 0.4, *B*_1_ = 100, *B*_2_ = 10, and the initial conditions (*S*(0), *E*(0), *I*(0), *V*(0), *T*(0)) = (2,0.7,0.2,0.1,1) × 10^3^, we have the basic reproduction number *R*_0_ = 1.3997 > 1, and the numerical results are depicted in Figures 2 and 3.  Figure 2 shows the control variables *u*_1_ and *u*_2_ when *R*_0_ > 1. By Figure 2, we see that, to minimize the number of infectious and latent individuals, the control *u*_1_ keeps the increasing trend during 5 days; during the remaining 15 days, it decreases to the lower bound. The control *u*_2_ is at the upper bound during 17 days; during the remaining 3 days, it decreases to the lower bound.  Figure 3 shows that the number of the susceptible individuals is higher, the numbers of the latent individuals, infectious individuals, and recovery individuals are lower, and the number of the vaccination individuals is with almost no change when controls are considered.

**Figure 4:**
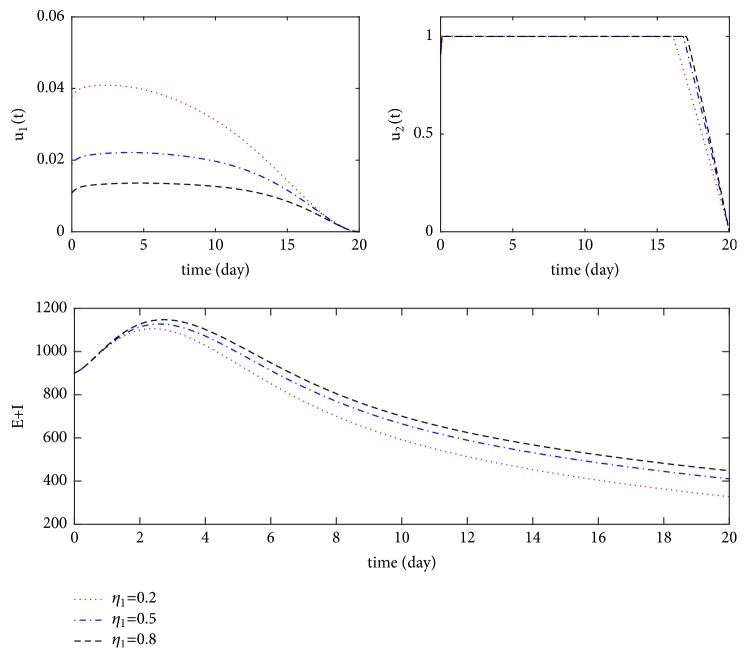
*E* + *I* with and without control for *η*_1_ = 0,2, 0.5,0.8, respectively.

We discussed the influence of immune loss rate on the spread of disease. That is, we discuss the influence of *η*_1_ on the basic regeneration number *R*_0_ in the following. Because of

(36) $$\begin{array}{c} \frac{\partial R_{0}}{\partial \eta_{1}}==\frac{\beta \alpha }{\left(\mu +\alpha \right)\left(\mu +d+\gamma \right)}\times \frac{pb}{{\left(\mu +\omega +\eta_{1}\right)}^{2}}>0, \end{array}$$

we know the basic reproduction number *R*_0_ increases as *η*_1_ increases. In order to control the basic regeneration number *R*_0_ less than 1, we need the immune loss rate *η*_1_ to satisfy *η*_1_ < *η*_1_^*∗*^, under the condition *αβb*(1 − *p*)/(*μ* + *α*)(*μ* + *d* + *γ*)(*μ* + *ω*) < 1 < *αβb*/(*μ* + *α*)(*μ* + *d* + *γ*)(*μ* + *ω*), where *η*_1_^*∗*^ = [(*μ* + *ω*)(*μ* + *α*)(*μ* + *d* + *γ*) − *αβb*(1 − *p*)](*μ* + *ω*)/(*αβb* − (*μ* + *ω*)(*μ* + *α*)(*μ* + *d* + *γ*)) > 0. Let *η*_1_ = 0,2, 0.5,0.8, respectively; other parameter values are the same as those in Figure 3. We obtain Figure 4. The higher the immune loss rate *η*_1_, the greater the number of infections and latent individuals. At the same time, higher immunization loss rate *η*_1_ indicates that the immunization control measures *u*_1_ are weakened. Accordingly, the treatment control measures *u*_2_ need to be strengthened.

**Figure 5:**
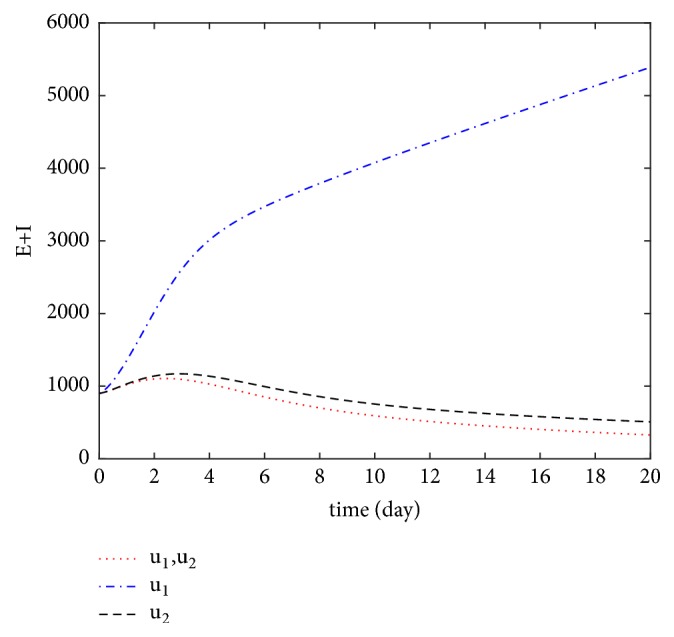
The number of *E* + *I* with different optimal control.

**Figure 6:**
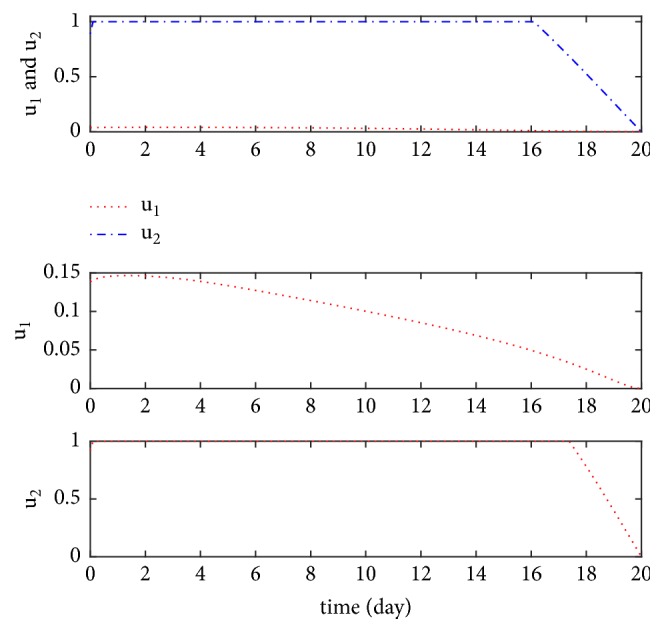
The optimal control variables *u*_1_^*∗*^ and *u*_2_^*∗*^ under different optimal control.

We also compared the number of infections and latent individuals under different control measures (see Figure 5). In Figure 5, the red dots indicate that control measures *u*_1_ and *u*_2_ are implemented simultaneously, the blue dots indicate that only control measure *u*_1_ is implemented, and the black dotted line indicates that only control measure *u*_2_ is implemented. The numerical simulation results show that the number of infections is minimal when the control measures *u*_1_ and *u*_2_ are implemented simultaneously. If only one control was implemented, the treatment control *u*_2_ would be more effective than vaccination control *u*_1_ in controlling the number of infectious and latent individuals. The trend of the controls *u*_1_ and *u*_2_ is displayed in Figure 6 under different control measures.

## Data Availability

No data were used to support this study.
